# A Novel Continuous Real-Time Vital Signs Viewer for Intensive Care Units: Design and Evaluation Study

**DOI:** 10.2196/46030

**Published:** 2024-01-05

**Authors:** Shiming Yang, Samuel Galvagno, Neeraj Badjatia, Deborah Stein, William Teeter, Thomas Scalea, Stacy Shackelford, Raymond Fang, Catriona Miller, Peter Hu, VS viewer study group

**Affiliations:** 1 Department of Anesthesiology University of Maryland School of Medicine Baltimore, MD United States; 2 Department of Neurology University of Maryland School of Medicine Baltimore, MD United States; 3 Department of Surgery University of Maryland School of Medicine Baltimore, MD United States; 4 Emergency Medicine University of Maryland School of Medicine Baltimore, MD United States; 5 United States Air Force Academy Colorado Springs, CO United States; 6 Department of Surgery Johns Hopkins University School of Medicine Baltimore, MD United States; 7 See Acknowledgments

**Keywords:** clinical decision-making, health information technology, intensive care units, patient care prioritization, physiological monitoring, visualization, vital signs

## Abstract

**Background:**

Clinicians working in intensive care units (ICUs) are immersed in a cacophony of alarms and a relentless onslaught of data. Within this frenetic environment, clinicians make high-stakes decisions using many data sources and are often oversaturated with information of varying quality. Traditional bedside monitors only depict static vital signs data, and these data are not easily viewable remotely. Clinicians must rely on separate nursing charts—handwritten or electric—to review physiological patterns, including signs of potential clinical deterioration. An automated physiological data viewer has been developed to provide at-a-glance summaries and to assist with prioritizing care for multiple patients who are critically ill.

**Objective:**

This study aims to evaluate a novel vital signs viewer system in a level 1 trauma center by subjectively assessing the viewer’s utility in a high-volume ICU setting.

**Methods:**

ICU attendings were surveyed during morning rounds. Physicians were asked to conduct rounds normally, using data reported from nurse charts and briefs from fellows to inform their clinical decisions. After the physician finished their assessment and plan for the patient, they were asked to complete a questionnaire. Following completion of the questionnaire, the viewer was presented to ICU physicians on a tablet personal computer that displayed the patient’s physiologic data (ie, shock index, blood pressure, heart rate, temperature, respiratory rate, and pulse oximetry), summarized for up to 72 hours. After examining the viewer, ICU physicians completed a postview questionnaire. In both questionnaires, the physicians were asked questions regarding the patient’s stability, status, and need for a higher or lower level of care. A hierarchical clustering analysis was used to group participating ICU physicians and assess their general reception of the viewer.

**Results:**

A total of 908 anonymous surveys were collected from 28 ICU physicians from February 2015 to June 2017. Regarding physicians’ perception of whether the viewer enhanced the ability to assess multiple patients in the ICU, 5% (45/908) strongly agreed, 56.6% (514/908) agreed, 35.3% (321/908) were neutral, 2.9% (26/908) disagreed, and 0.2% (2/908) strongly disagreed.

**Conclusions:**

Morning rounds in a trauma center ICU are conducted in a busy environment with many data sources. This study demonstrates that organized physiologic data and visual assessment can improve situation awareness, assist clinicians with recognizing changes in patient status, and prioritize care.

## Introduction

Clinicians working in intensive care units (ICUs) must be able to see, understand, and respond quickly to the complex and ever-changing clinical environment of the ICU. They need to be able to collect, analyze, and interpret what is happening and what it means [1]. Situational awareness is essential for ICU clinicians to provide safe and effective care to their patients. When clinicians have good situational awareness, they are better able to identify and respond to changes in their patients’ condition and to coordinate care with other members of the health care team. However, clinicians are immersed in a cacophony of alarms and a relentless onslaught of data. Within this frenetic environment, clinicians make high-stakes decisions using multiple data sources and are often oversaturated with information of varying quality. While modern hospitals are equipped with bedside monitors collecting various physiological data in a real-time, continuous, and automated way, these data are not always easily accessible remotely or available to be viewed as a continuous trend [2]. The enormous amount of unprocessed data adds an additional burden on ICU clinicians who work in a dynamic environment with voluminous decision-making requirements. Traditional bedside monitors only show a single patient’s instantaneous (static) vital signs (VS) data, limiting the clinician’s scope to view a patient’s physiological trajectory within a clinically meaningful period of time. Clinicians must rely on separate nursing charts—handwritten or electronic—to review a patient’s physiological status. Moreover, auditory alarms often cause “alarm fatigue” instead of increasing situational awareness [3]. Many bedside monitors only display 1 or 2 patients’ information; the ability to view an entire unit or ward allows a clinician to prioritize attention to those in most need of critical care support [4]. Improved visualization of patient information may help clinicians cope with information overload in critical care settings by improving situational awareness and supporting clinical decision-making [5]. An automated physiological data-organizing and information-summary system that presents aggregated information from multiple data sources while providing at-a-glance summaries of clinical data can assist ICU clinicians with prioritizing care for multiple patients.

Developed initially for use in aircraft transporting multiple patients who are critically ill, this VS viewer has 2 outcomes of direct and important clinical applicability. First, the VS viewer can provide clinicians with the capability to monitor individual patient trends, improving overall decision-making. Since patients in the ICU require multiple life support treatments to ensure ideal long-term outcomes, improved display of VS patterns could improve patient assessment and clinical decision-making. Second, the VS viewer system allows remote monitoring of groups of patients through a display that provides clinicians with the ability to quickly identify patients in need of rapid intervention. The objective of this work is to evaluate the use of a VS viewer in ICUs at a high-volume level 1 trauma center. We hypothesized that clinicians would subjectively report improved situational awareness and enhanced ability to make clinical decisions with the use of a VS viewer.

## Methods

### Data and System Design

In the ICUs of the University of Maryland Medical System, GE Marquette Solar 7000/8000 (General Electric) patient VS monitors are networked to provide a collection of real-time patient VS data streams. Each patient monitor collects real-time 240 Hz waveforms and 0.5 Hz trend data, which are transferred through the secure intranet to a dedicated BedMaster server (Excel Medical Electronics) and archived [6]. To increase the system’s availability and reliability, a triple-redundant design was used, in which 3 BedMaster servers were used in parallel to collect data from all bed units [7]. Physiological data collected through this system, when they are displayed on the GE Marquette monitor, include electrocardiographic, photoplethysmographic, carbon dioxide, arterial blood pressure, and intracranial pressure (ICP), among others. Trends include heart rate (HR), respiratory rate, temperature, oxygen saturation, end-tidal carbon dioxide, and ICP, among many others. This information provides continuous VS data that relays important physiological information regarding brain perfusion, cardiac stability, overall tissue perfusion, and respiratory status.

During the design of the VS viewer for ICU, our goal was to create a novel physiological data displayer that can reduce ICU clinicians’ workload, enhance clinical decision-making, and improve communication in a noisy and confined ICU environment. To achieve the goal, we considered the factors of usability and patient safety, which can be closely related in this application. For usability, current bedside monitors often suffer from insufficient time windows to display physiological trends, a lack of clear indications of patients’ physiological status, and a lack of overview of multiple patients for prioritizing [4]. To enhance the clinicians’ efficiency while maximizing patients’ safety, we adopted the following design strategies: First, the viewer should reduce the information overload for clinicians to access patients’ physiological data, current or past, individual or group [8-10]. Second, it should be compatible with the existing patient monitor system so that clinicians can reuse their existing knowledge about the monitor, which may increase the acceptance of the VS viewer [8]. Third, in the user experience design, the viewer should place the user in control [11]. It should use simple colors and graphs to convey efficient information while still providing detailed data for advanced users to access with simple operations [12]. Fourth, the viewer should have reasonable reliability for patients’ safety. Redundance was introduced in the design for key components in the system, such as the data collection, database, and web server [7].

The VS viewer adopted a client-server architecture. The server handles 2 types of clients: the bedside monitors and the users. It receives and persists in real-time physiologic data that are transmitted from the bedside monitors. A database records each bedside VS value, bed name, and timestamp. The server also responds to users’ requests for viewing data within a given time frame. To continuously present the latest data to the user with low latency, the VS viewer uses the asynchronous Javascript and XML technique to pull the most recent data from the database every minute [13]. Such a method allows the VS viewer to automatically redraw all VS trajectories without refreshing the entire viewing page.

**Figure 1:**
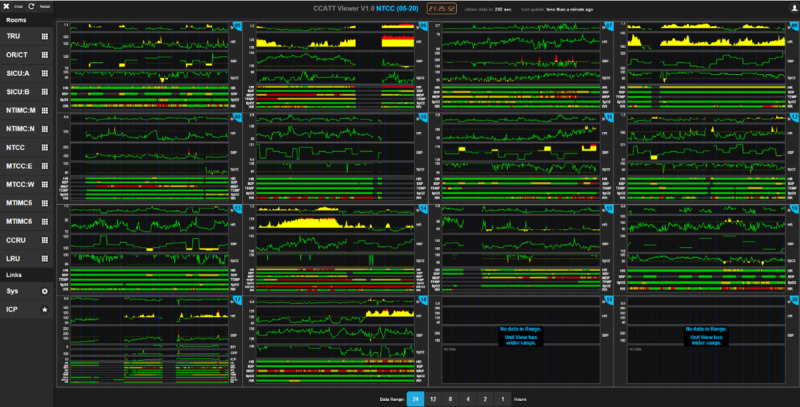
The vital sign viewer in the “group” mode, with a default 24-hour display.

The VS viewer provides a rich interface for data monitoring, exploration, and recording. Data are depicted according to each clinical area of operations, such as the trauma resuscitation unit or emergency department, operating room, computed tomography suite, and individual critical care units. Figure 1 demonstrates the grouping of bed units. On the left panel, a list of all groups can be used as a shortcut to bed units. Selecting a specific unit, a default 24-hour view is displaced for shock index (SI=HR/systolic blood pressure), HR, systolic blood pressure, ICP, cerebral perfusion pressure, brain trauma index, and end-tidal carbon dioxide concentration. If ICP data are not collected, the space is used to plot the next available VS, optimizing the view.

**Figure 2:**
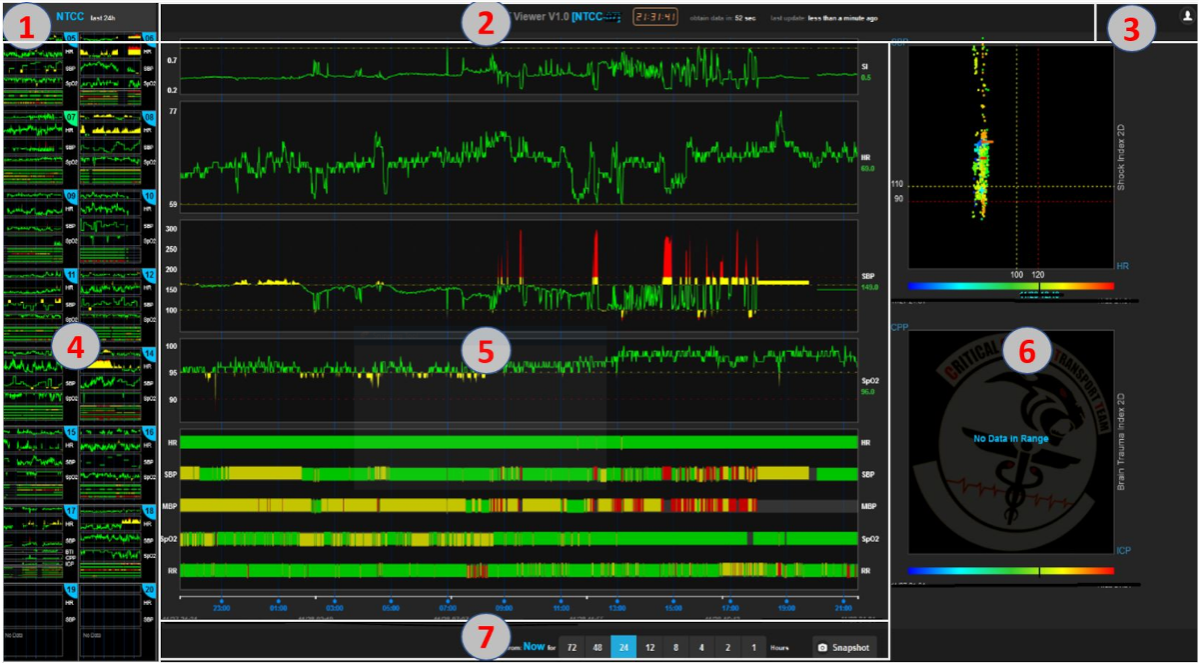
Vital sign (VS) viewer in the “unit” mode, with default 24-hour display. Labeled area 1: navigation menu to other room groups. Area 2: title information for room name, current time, and the next update time. Area 3: user portal. Area 4: list of beds in the same group with their current VS thumbnails. Area 5: the main area to display selected room VS trajectories and the summarization with color-coded patterns. Area 6: diagnostic tools for 2D scatter plots of shock index (=heart rate/systolic blood pressure) and brain trauma index (=intracranial pressure/cerebral perfusion pressure). Area 7: functional buttons for selecting various time ranges for viewing.

When a bed is selected, a page for this bed (unit view) is displayed. Figure 2 demonstrates the structure of the information. The page is partitioned into multiple areas for navigation, viewing, and tools. Its center is assigned for presenting the selected patient’s physiologic data in a time frame (up to 72 hours). VS trajectories are stacked vertically in order of predefined importance. The bottom is reserved for plotting bar segments of all VS that summarize the colored warnings without showing the value changes. This provides a summary of all available VS trends in a condensed space, which could be used to view the physiological stability of the patient over time. To provide an at-a-glance view of other rooms in this group, the left panel lists all the rooms in the current group and updates their VS trajectories in real time. The color-coded warning in the thumbnails enhances situation awareness even when the users are focusing on 1 patient.

**Figure 3:**
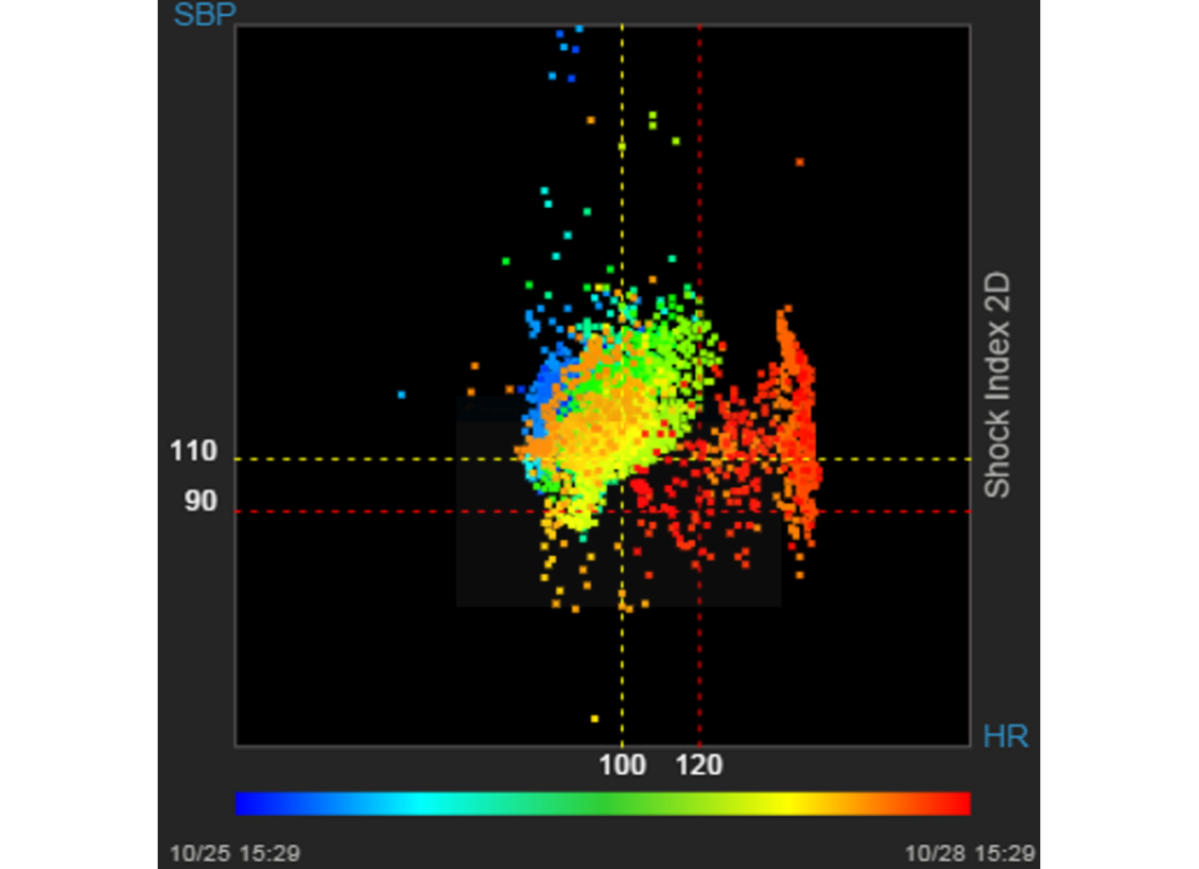
An example 2D shock index plot. The colored scatter plot shows the change in shock index (heart rate/systolic blood pressure) from past (blue) to recent (red), thereby depicting a 3-day change in worsening shock index.

The VS viewer has additional diagnostic tools. For example, SI is a commonly used blood transfusion diagnosis tool [14]. The VS viewer adds a 2D SI diagram to show a changing trajectory (Figure 3). To present the temporal information, a heat map is plotted, ranging from blue (cold) to red (warm); blue colors represent past events, whereas red colors represent current data trends. Similarly, the brain trauma index (which is ICP or cerebral perfusion pressure) can also be visualized in the 2D plot [15].

### Clinical Thresholds

Colored warnings are an effective means to gain a clinician’s attention and may be more effective than audible alarms, especially in a noisy, busy, and confined environment [16]. In the VS viewer, VS trajectories with colors may be viewed to highlight the sections where the VS are outside of normal clinical thresholds. For example, too low or too high HR segments are displayed differently from normal HR. Clinical thresholds for VSs were developed after surveying 47 clinicians (24 medical doctors, 18 registered nurses, and 5 respiratory therapists). Among them, 36 clinicians were from the University of Maryland, Baltimore, and 11 from the University of Cincinnati. After the survey was completed, a team of clinicians met to review the results to reach a consensus on the viewer’s opinion of their visual appearance. Multimedia Appendix 1 summarizes the optional threshold distributions for some important VS. Based on these threshold values, a consensus set of color-coded cutoffs was determined (Multimedia Appendix 2). These values were set as fixed parameters under consideration of a simplified and consistent user interface.

### Survey Design

Clinicians who were scheduled to work in the ICU or on the trauma teams were contacted and trained on how to use the VS viewer. Once trained, ICU and team clinicians were asked to participate in the study. Clinicians were surveyed anonymously from Tuesday to Friday and were asked to conduct rounds normally, using data reported from nurse charts and briefs from fellows to inform their clinical decisions. None of those clinicians participated in the design of the VS viewer. A total of 2 questionnaires were designed to collect clinicians’ opinions about a patient’s condition and satisfaction with the VS viewer. Clinicians were given a preview survey upon their assessment and formulation of their plan for each patient after traditional rounds and before accessing the viewer. Immediately following the completion of the pre-view survey, the VS viewer was presented to the clinicians on a tablet, displaying the patient’s past physiologic data visualized and summarized for up to 72 hours. After reviewing the viewer for up to 1 minute, clinicians completed the postview questionnaire. In both questionnaires, the clinicians answered questions regarding the patient’s stability, status, and need for a higher or lower level of care. In the post-view questionnaire, clinicians were also asked if they intentionally planned to implement any of the following interventions after seeing the viewer: (1) changing any current medications, (2) ordering additional medications, (3) ordering additional diagnostic tests, (4) changing ventilation settings, (5) ordering additional labs, (6) physically reexamining this patient, (7) providing fluid bolus, or (8) providing a blood transfusion.

### Statistical Methods

A participant’s perceiving of the VS viewer’s usefulness is represented by a vector consisting of the percentage of the 5 categories (strongly agree, agree, neutral, disagree, and strongly disagree) that he or she assigned to the question “the viewer enhanced my understanding of the patient’s condition.” We used the Ward method, a hierarchical clustering method, with Manhattan distance to group the participants based on their ratings to the question “the viewer enhanced my understanding of the patient’s condition” [17,18]. Between those clusters, we compared the participants’ opinion changes on the patients’ conditions in 7 questions (Table 1) before and after using the viewer. The chi-square test was used to compare percentage differences.

**Table 1:** The number of opinion changes for 7 questions (Q1-Q7) before and after seeing the viewer, with respect to the 5 clustered user types.

Questions^a^	Total changes, n (%)	Unique participants, n (%)	C1, n	C2, n	C3, n	C4, n	C5, n	Like (C1 and C2), n (%)	Dislike (C3, C4, and C5), n (%)
Q1	129 (14.2)	16 (66.7)	46	31	10	42	0	77 (59.7)	52 (40.3)
Q2	112 (12.3)	15 (62.5)	38	34	8	32	0	72 (64.3)	40 (35.7)
Q3-6	145 (16)	18 (75)	58	54	3	30	0	112 (77.2)	33 (22.8)
Q7	92 (10.1)	17 (70.8)	20	32	9	31	0	52 (56.5)	40 (43.5)

^a^Please refer to Textbox 1 for the question.

**Questions.**

- Q1: Having reviewed the last 24 hours of information during rounds and before and after seeing the 24-hour viewer, do they feel that in the past 24 hours the patient has shown evidence of (a) infection, (b) hemodynamic instability, (c) uncontrolled bleeding, or (d) respiratory deterioration?
- Q2: Over the past 24 hours, has the patient’s condition (a) improved significantly, (b) improved slightly, (c) unchanged, (d) deteriorated slightly, or (e) deteriorated significantly?
- Q3: Can the patient be transferred to a lower level of care?
- Q4: Can the patient be transferred to a higher level of care?
- Q5: Does the patient have a traumatic brain injury?
- Q6: Did the patient have intracranial pressure problems in the past 24 hours?
- Q7: Due to the viewer, do they plan for any changes in interventions, including (a) changing any current medications, (b) ordering additional medications, (c) ordering additional diagnostic tests, (d) changing ventilation settings, (e) ordering additional labs, (f) physically reexamining this patient, (g) providing a fluid bolus, or (h) providing a blood transfusion?

Note: These are the questions referenced in Table 1.

### Ethical Considerations

The study has been approved by the institutional review board of the University of Maryland School of Medicine (HP-00063086).

## Results

### Survey Collection

**Figure 4:**
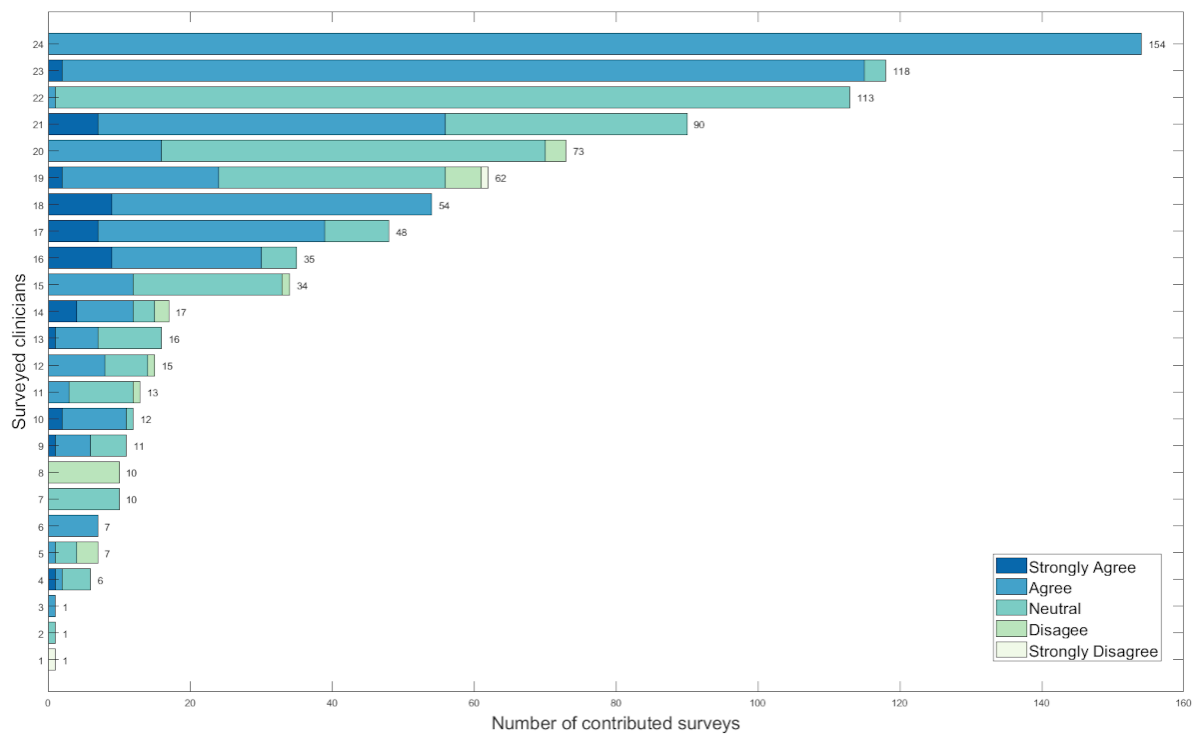
Distribution of each participant’s rating on if the viewer enhanced his or her understanding of the patient’s condition during a round.

From February 2017 to June 2017, the survey team followed clinicians who agreed to take the surveys. A total of 908 surveys were collected from 24 participants with unbalanced proportions. Among the 908 rounds, 48 (5%) were patients who were newly admitted, and 860 (95%) were not. When asked if the VS viewer enhanced their understanding of the patient’s condition, clinicians strongly agreed 45 (5%) times, agreed 514 (56.6%) times, were neutral 321 (35.4%) times, disagreed 26 (2.9%) times, and strongly disagreed 2 (0.2%) times. Figure 4 lists the total surveys each participant contributed and the proportions of ratings on whether the viewer enhanced his or her understanding of the patient’s condition during a round.

Results show that physicians’ clinical assessments and plans could be influenced by viewing the VS viewer for 1 minute or less, indicated by a “yes” answer to at least 1 of the 8 questions (Q7 in the survey). Of the 908 rounds, a total of 92 (10.1%) rounds had at least 1 “yes” as planning on some changes to the interventions. The most common change was (Q1) changing current medications (36/908, 4%). The next most common changes were (Q6) physically reexamining the patient (31/908, 3.4%), (Q2) ordering additional medications (20/908, 2.2%), and (Q7) providing a fluid bolus (20/908, 2.2%).

**Figure 5:**
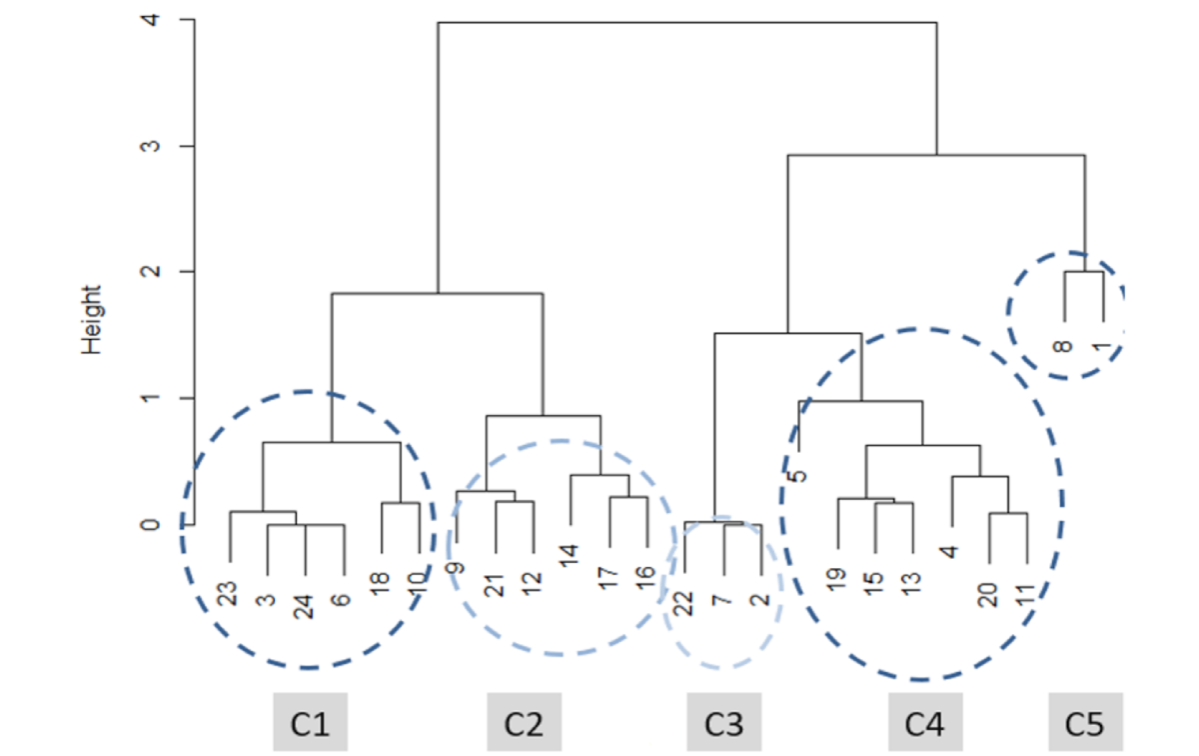
Clusters of participants with similar feelings about the viewer. In total, 24 unique participants are grouped into 5 groups, corresponding to “strongly favor,” “favor,” “neutral,” “dislike,” and “strongly dislike.”.

We used the Ward method with Manhattan distance to group the participants based on their ratings to the question “the viewer enhanced my understanding of the patient’s condition” [17]. For example, 1 participant contributed 62 surveys and rated 2 “strongly agree,” 22 “agree,” 32 “neutral,” 5 “disagree,” and 1 “strongly disagree.” The vector of percentages (0.03, 0.35, 0.52, 0.08, and 0.02) represents the overall rating that this participant had about the viewer. The 24 participants were clustered into 5 groups, as shown in Figure 5. The 5 groups correspond to the participants who are mostly in favor (C1) of the viewer to those least in favor (C5). There are 6 in C1, 6 in C2, 3 in C3, 7 in C4, and 2 in C5, which shows a very balanced grouping, with half of the participants in the C1 and C2 groups and the other half in the other 3 clusters. This shows that the sampled rounds were done by participants with almost similar proportions of different attitudes toward the viewer. In other words, the survey team sampled the rounds randomly enough so that the collected data were not biased by participants with certain preexisting feelings about the viewer.

### Comparisons

We analyzed the opinion changes before and after seeing the viewer, regarding the patient’s stability, status, and need for a higher or lower level of care. Instead of summarizing the total changes in opinions, we compared them with respect to the clusters of user types. The participants who were “neutral” (C3) or “strongly dislike” (C5) had low numbers of opinion changes for all 7 questions. Those who were in clusters C1, C2, and C3 had more numbers of opinion changes (Table 1). For simplicity, we can further group the participants into 2 types: those who liked the VS viewer (C1 and C2) and those who disliked it (C3, C4, and C5). The clinicians who liked the VS viewer had a higher rate of changed opinions than those who disliked the VS viewer regarding Q1 to Q6 (Q1: 59.7% vs 40.3%, Q2: 64.3% vs 35.7%, and Q3-6: 77.2% vs 22.8%). When asked if they planned for any changes for interventions (Q7), there was no significant difference between the 2 major groups of clinicians (56.5% vs 43.5%, *P*=.10).

## Discussion

### Principal Results

With the development of sensor and computing technologies, vast amounts of high-quality, continuous electronic data, including VS, alarms, and clinical interventions, are collected at the bedside. Those data have the potential to provide an unprecedented view of dynamic physiologic responses to injury, illness, and treatments. Therefore, data gathered from bedsides could assist clinicians in care planning and decision support. However, massive amounts of data that are not well organized or presented still create a barrier for clinicians making full use of them in a busy resuscitation or intensive care environment. Bedside monitors often only display instantaneous readings or a short strip of recent physiologic VS for diagnosis. Clinicians need to rely on separate nursing charts, handwritten or electronic, to review a patient’s developing conditions. The VS viewer, which automates physiological data by displaying clear color-coded trends, presents aggregated information from multiple data sources, provides at-a-glance summaries of clinical data, and assists with the prioritization of care for multiple patients.

The use of the VS viewer was subjectively assessed with 908 observations from clinicians working in ICUs at a high-volume level 1 trauma center. Clinicians generally perceived the use of the VS viewer favorably, as evidenced by survey data. The VS viewer was originally developed for the United States Air Force Critical Care Air Transport Teams [19,20]. Critical Care Air Transport Teams transport up to 3 patients who are critically ill in the back of the aircraft, allowing trauma surgeons to perform far-forward damage control surgery, knowing that these patients could be quickly transported rearward with full support. This rapid transport of complex patients with multisystem trauma, shock, burns, and respiratory failure who are in hemodynamic flux requires continual resuscitation, stabilization, advanced care, and life-saving interventions during air transport; however, currently available advanced ICU monitoring systems suitable for the needs of such patients were developed for use in stable, hospital-based settings, not in the crowded, noisy, vibrating, and sometimes frankly jolting environment of air evacuation or long-distance air transport. The noise levels, confined space, limited access to patients, vibration, and overall limited patient visibility make using a VS viewer advantageous in such a setting. Such technology can also be valuable in enhancing emergency medical personnel’s decision-making for initial triage. While traditional VS are useful in guiding prehospital care and triage, they represent isolated points in time, and trends and fluctuations in vitals may not be apparent.

In this study, we set the clinical thresholds for colored warnings to be uniform across all beds. This was to make the user interface simplified and more consistent during a survey. Additionally, a set of predefined thresholds from a group of experienced clinicians could be a useful out-of-the-box feature when the VS viewer is deployed in the field. That said, the clinical thresholds could be personalized for each bed. For example, if the bedside monitor allows alarm threshold settings, such settings could be used as the colored warning thresholds in the VS viewer for each bed.

The VS viewer has expanded from ICUs to trauma resuscitation units, operating rooms, neuro ICUs, and pediatric ICUs at the University of Maryland Medical Center. In 2020, during the COVID-19 pandemic, it was deployed to monitor 150 beds in biocontaminated units to reduce the risk of infection and improve efficiency for clinicians in treating their patients.

### Innovations

The VS viewer is a multipatient physiological monitor. To the best of our knowledge, we could not find any articles that describe a viewer system with a similar design. In a comprehensive review by Waller et al [5], a total of 17 information displays in ICU settings were designed for specific disease states or body systems, such as cerebral perfusion monitoring for individual patients or monitoring for arterial blood gas trends. The novel user interface presented in this study was designed with the aim of conveying information more efficiently to ICU clinicians in a noisy, confined, and busy environment. It uses color-coded warnings to indicate a patient’s status and highlight data that needs attention. The side panel provides a peek at the physiological status of other patients, which can help clinicians keep an eye on other patients even if their attention is focused on a single patient. It uses advanced web front-end techniques to hide large quantities of data behind simple line charts and reveal them when needed.

### Clinical Impact

The use of the VS viewer can have several possible influences on clinical assessment and plans. It can help clinicians quickly recognize critical changes in the patient’s physiologic status and provide early interventions to prevent further deterioration. The VS viewer can potentially improve patient outcomes by providing clinicians with a concise overview of key information, reducing cognitive load and errors, and improving compliance with evidence-based safety guidelines [12]. It may also help to improve communication efficiency within the ICU team by providing easy access to a shared platform of patient longitudinal data. It can reduce the workload of the ICU team by automating routine tasks such as extracting data from nursing charts.

To prioritize care in high-volume ICUs, intensive care clinicians must be able to rapidly identify physiological events and the need for intervention. The VS viewer can help organize a large amount of data in a busy, noisy ICU environment where close monitoring of patients who are critically ill is essential to detect potentially harmful physiological trends. The presentation of data with temporal, color-coded patterns, and the ability of the VS viewer to provide at-a-glance data for entire units is advantageous for clinicians working in high-volume ICUs.

The color-coded patterns may reduce the “alarm fatigue” issue in noisy ICUs. The noise burden is common in modern physiologic monitoring systems and has been recognized as a critical patient safety concern in the hospital care setting [21-23]. In noisy environments, such as ICUs, helicopter transportation, or aeromedical evacuation, loud and continuous alarms could reduce their specificity in getting clinicians’ responses. Another issue with audible alarms is that they are transient and cannot be replayed once they are gone. While the visual alert patterns could show the longitudinal patterns of physiologic change.

### Related Work

The VS viewer with organized and easy-access information could be part of the effort to build the smart ICU or the tele-ICU. The concepts of smart ICU and tele-ICU aim to maximize the use of bedside clinical expertise in assessing and treating patients by providing integrated monitoring and actionable information [24-26]. A survey study of 86 ICU staff in a German university hospital summarized that health providers expect ICU monitoring could be improved by reducing false alarms, using wireless sensors and mobile devices, preparing for the use of AI, and enhancing the digital literacy of ICU staff [27,28]. The VS viewer could be used in both centralized and decentralized architectures of tele-ICU for extending coverage and facilitating patient transfer between hospitals because of its flexible configuration of grouping ICU beds virtually [29]. By making essential clinical information available remotely, the VS viewer allows clinicians to provide care plans when on-site support is infeasible or limited [30,31]. It may potentially reduce exposure to contagious diseases and, hence, increase patient safety.

With continuous physiologic data and other clinical information, the VS viewer has the ability to process real-time data into predictive algorithms, which is also desired for tele-ICU [30]. Beyond being a plain displayer, the VS viewer could embed risk-prediction algorithms that use continuous VS as inputs and may promote more efficient interventions to reduce ICU risk [31]. For example, ICU mortality prediction [32,33], secondary insults after severe brain traumatic injury [34], needs for transfusion [35,36], and neurologic decline in the ICU [37] are reported to have good predictive performances by using variables derived from continuous VS. We have also shown that using risk scores calculated from continuously measured VS, patients requiring endovascular resuscitative interventions can be identified with high accuracy [38]. Moreover, the VS viewer could serve as a platform for predictive model diagnosis by providing clinicians with explainable artificial intelligence [39]. With patient VS data, we can use the Shapley Additive exPlanations algorithm to calculate each variable’s contribution to the prediction result [40]. Therefore, the clinicians would know not only the prediction but also the contribution of each variable to the prediction. Such information may help clinicians make more personalized care plans.

### Limitations

There are limitations to this work that are worth noting. We collected data from a large number of ICU clinicians compared to trauma team clinicians. Trauma team clinicians are surgeons responsible for the same patient throughout the entire length of stay, regardless of the acuity of the patient. ICU clinicians are intensivists and are only responsible for patients in the ICU. Hence, disparities between both groups of clinicians are inevitable, as each group has different clinical perspectives and patient workloads. As occurs in nearly all survey work, response rates and receptiveness to the surveys varied. Some clinicians were more amenable to being surveyed compared to others. In the collected forms, there were more surveys from some clinicians than from others. To reduce this potential bias, we clustered the participants based on their overall rating on each round, from which we estimated each participant’s a priori attitude toward using this viewer. The results show that there was a balanced “favoring” and “non-favoring” of using this viewer.

We only evaluated the viewer based on clinicians’ satisfaction and efficiency (potential changes in interventions before and after seeing the viewer). In future studies, randomized controlled trials can be designed to analyze the viewer’s impact on patients’ outcomes and safety [12].

### Conclusions

We designed, implemented, and evaluated an automated physiologic data organizer and visualization platform. It provides at-a-glance summaries and assists with prioritizing care for multiple patients. The VS viewer demonstrates a method to assemble large quantities of data from multiple sources and represents trends in each patient’s condition with simple color codes, greatly improving situational awareness. It has the potential to be used in en route care, hospitals with multiple branches, and understaffed hospitals in remote areas. The survey shows that organized physiologic data and visual assessment could assist clinicians in recognizing changes in patient status and prioritizing care.

## Appendix

Multimedia Appendix 1 Surveyed thresholds for heart rate, systolic and diastolic blood pressure, blood oxygen saturation, and temperature.

Multimedia Appendix 2 VS Viewer color coding threshold values.

## Abbreviations

HR: heart rate
ICP: intracranial pressure
ICU: intensive care unit
SI: shock index
VS: vital signs
