# Supplementary material for: A Novel Continuous Real-Time Vital Signs Viewer for Intensive Care Units: Design and Evaluation Study
Source: JMIR Hum Factors. 2024 Jan 5;11:e46030. doi: 10.2196/46030 (PMC10799282; doi:10.2196/46030)
Supplement: Multimedia Appendix 1 [file humanfactors_v11i1e46030_app1.docx]

| **VS** | **High – Red High Lower Limit** | **Yellow High** | | **Green** | | **Yellow Low** | | **Low – Red Low Upper Limit** |
| --- | --- | --- | --- | --- | --- | --- | --- | --- |
|  |  | **Upper Limit** | **Lower Limit** | **Upper Limit** | **Lower Limit** | **Upper Limit** | **Lower Limit** |  |
| **HR** |  |  |  |  |  |  |  |  |
| Mean | 121 | 119 | 100 | 100 | 62 | 59 | 50 | 49 |
| Median | 120 | 119 | 101 | 100 | 60 | 59 | 51 | 50 |
| SD | 8.68 | 8.59 | 4.26 | 4.90 | 5.98 | 5.09 | 4.83 | 4.57 |
| N | 19 | 17 | 17 | 17 | 17 | 17 | 17 | 19 |
| **SBP** |  |  |  |  |  |  |  |  |
| Mean | 175 | 174 | 147 | 145 | 103 | 102 | 88 | 86 |
| Median | 180 | 179 | 150 | 149 | 101 | 100 | 90 | 89 |
| Mode | 180 | 179 | 160 | 159 | 100 | 99 | 81 | 80 |
| SD | 14.99 | 15.43 | 13.08 | 13.07 | 8.60 | 8.55 | 6.59 | 5.71 |
| N | 17 | 16 | 16 | 15 | 15 | 16 | 17 | 18 |
| **DBP** |  |  |  |  |  |  |  |  |
| Mean | 103 | 102 | 86 | 85 | 56 | 56 | 43 | 43 |
| Median | 100 | 103.5 | 88 | 87 | 60 | 59 | 41 | 40 |
| SD | 12.40 | 13.41 | 14.28 | 14.28 | 9.05 | 9.62 | 7.54 | 7.55 |
| N | 14 | 12 | 12 | 12 | 12 | 13 | 13 | 14 |
| **SaO2** |  |  |  |  |  |  |  |  |
| Mean |  |  |  | 100 | 93.9 | 92.9 | 89.3 | 88.3 |
| Median |  |  |  | 100 | 94.5 | 93.5 | 90 | 89 |
| SD |  |  |  | 0.00 | 1.77 | 1.77 | 2.37 | 2.37 |
| N |  |  |  | 14 | 14 | 14 | 14 | 14 |
| **Temp** |  |  |  |  |  |  |  |  |
| Mean | 39 | 39 | 38 | 38 | 36 | 36 | 35 | 35 |
| Median | 39 | 39 | 38 | 38 | 36 | 36 | 35 | 35 |
| SD | 1.11 | 1.08 | 0.47 | 0.46 | 0.64 | 0.48 | 0.70 | 0.66 |
| N | 17 | 13 | 13 | 13 | 13 | 12 | 12 | 16 |

DBP = diastolic blood pressure; SaO2 = blood oxygen saturation; SD = standard deviation.
