# Supplementary material for: A Novel Continuous Real-Time Vital Signs Viewer for Intensive Care Units: Design and Evaluation Study
Source: JMIR Hum Factors. 2024 Jan 5;11:e46030. doi: 10.2196/46030 (PMC10799282; doi:10.2196/46030)
Supplement: Multimedia Appendix 2 [file humanfactors_v11i1e46030_app2.docx]

| **VR** | **Red** | **Yellow** | **Green** |
| --- | --- | --- | --- |
| HR | <50 or >120 | 50-59 or 100-119 | 59-100 |
| SBP | <81 or >180 | 81-99 or 160-179 | 100-160 |
| DBP | <41 or >100 | 41-59 or 91-99 | 60-90 |
| MBP | <51 or >90 | 51-59 or 71-89 | 60-70 |
| NSBP | <81 or >180 | 81-99 or 160-179 | 100-159 |
| NDBP | <41 or >100 | 41-59 or 91-99 | 60-90 |
| NMBP | <51 or >90 | 51-59 or 71-89 | 60-70 |
| SpO2 | <90 | 90-95 | 95-100 |
| “PULSE RATE” | <90 | 90-94 | 94-100 |
| TEMP | <35 or >39 | 35-36 or 38-39 | 36-38 |
| RR | <8 or >30 | 8-9 or 26-30 | 10-25 |
| ICP | >30 | 20-30 | 0-19 |
| EtCO2 | <20 or >50 | 20-30 or 40-50 | 30-40 |
| SI | >1.2 | >0.9 | <0.9 |
| CPP | <50 | 50-60 | >60 |
| BTI | <2 | 2-3 | >4 |

EtCO2 = end-tidal carbon dioxide; MBP = mean blood pressure; NDBP = non-invasive DBP; NMBP = non-invasive MBP; NSBP = non-invasive SBP; RR = respiratory rate; SpO2 = blood oxygen saturation (pulse oximetry).
